# The application and challenges of immune checkpoint inhibitors in lung cancer therapy

**DOI:** 10.3389/fimmu.2026.1811169

**Published:** 2026-04-20

**Authors:** Bingbing Li, Yuning Ren, Xiaoling Zhang

**Affiliations:** 1 Key Laboratory of Organ Regeneration and Transplantation of Ministry of Education, First Hospital of Jilin University, Changchun, China; 2 National-Local Joint Engineering Laboratory of Animal Models for Human Disease, First Hospital of Jilin University, Changchun, China

**Keywords:** immune checkpoint inhibitors (ICIs), lung cancer therapy, non-small cell lung cancer (NSCLC), small cell lung cancer (SCLC), tumor microenvironment (TME)

## Abstract

Lung cancer remains a leading cause of global cancer-related mortality, with most patients diagnosed at advanced stages. The emergence of immune checkpoint inhibitors (ICIs), specifically targeting PD-1/PD-L1 and CTLA-4 pathways, has revolutionized the treatment landscape by restoring anti-tumor immune responses. Currently, ICIs are integrated into clinical practice across all stages of lung cancer, administered either as monotherapy or in combination with chemotherapy, radiotherapy, and anti-angiogenic agents. Despite significant survival benefits, several critical challenges persist. First, primary and acquired resistance, driven by tumor microenvironment (TME) immunosuppression, impaired antigen presentation, and aberrant signaling, limits long-term efficacy. Second, existing biomarkers like PD-L1 expression and tumor mutational burden (TMB) face limitations due to insufficient standardization and spatiotemporal heterogeneity. Furthermore, immune-related adverse events (irAEs) across multiple organ systems necessitate vigilant clinical management and occasionally treatment discontinuation. This review systematically evaluates the research progress and clinical applications of ICI therapy in lung cancer. We highlight the therapeutic heterogeneity observed across different histological types, including non-small cell lung cancer (NSCLC) and small cell lung cancer (SCLC), with a specific focus on the management of brain metastases (BMs). Additionally, the article discusses varying response patterns within specific patient subgroups, such as geriatric patients and individuals with differing smoking or performance statuses. By synthesizing data on both favorable therapeutic outcomes and the spectrum of irAEs, we emphasize the necessity of personalized immunotherapy. Ultimately, this review looks forward to future advancements aimed at enhancing the precision and safety of lung cancer treatment, providing a roadmap for more tailored clinical interventions.

## 1 Introduction

**Figure 1:**
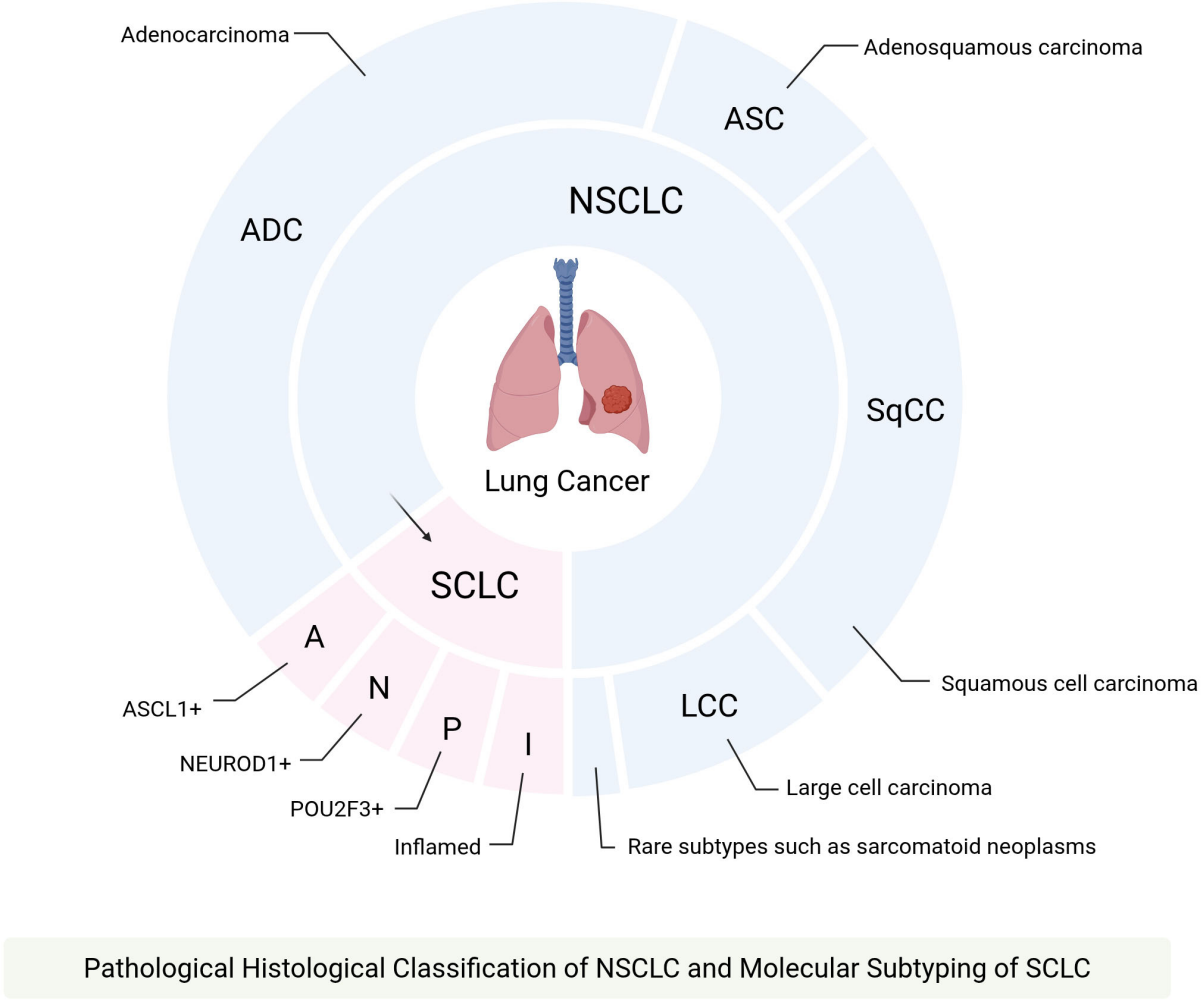
Primary subtypes of lung cancer. The pathological histological classification is the fundamental clinical categorization for NSCLC, primarily encompassing ADC, SqCC, adenocarcinoma with squamous differentiation (ASC), and LCC. Among these, ADC is the most prevalent subtype, followed by SqCC, while ASC exhibits components of both ADC and SqCC. SCLC is subdivided into four molecular subtypes based on molecular differences and inflammatory characteristics: SCLC-A, SCLC-N, SCLC-P, and SCLC-I. Additionally, NSCLC can transform into SCLC, a phenomenon primarily observed in patients with EGFR mutations but also seen in those with ALK, RET, KRAS, and other mutation types, the core molecular basis for this transformation is the co-inactivation of TP53/RB1.

Lung cancer is the second most common cancer worldwide and has the highest mortality rate of all cancers. SCLC accounts for around 15% of cases, while NSCLC makes up around 85% of lung cancer diagnoses (1). NSCLC includes several subtypes, primarily including adenocarcinoma (ADC), squamous cell carcinoma (SqCC), adenosquamous carcinoma (ASC) and large cell carcinoma (LCC), with adenocarcinoma being the most prevalent (2). Almost all SCLC patients exhibit dual loss of function in the tumor suppressor genes TP53 and RB1, which is a characteristic that strictly distinguishes SCLC from NSCLC (3) (**Figure 1**). Treatment requires consideration of disease stage, tumor genetic characteristics, and the patient’s physical condition. Diverse approaches include surgery, radiotherapy, chemotherapy, targeted therapy, and immunotherapy (4). Lung cancer development may be influenced by smoking, genetics, and air pollution, with smoking being the primary risk factor; non-smokers generally have a better prognosis (5).

Lung cancer is a highly heterogeneous malignancy, characterized by the rapid proliferation of tumor cells and a complex immune microenvironment. Even after complete surgical resection, many patients experience metastasis and recurrence. Lung cancer exhibits the highest incidence of brain metastasis among all solid tumors, carries a poor prognosis and is one of the leading causes of death in lung cancer patients. It is estimated that 10-30% of NSCLC patients will develop brain metastases (BMs) during disease progression (6), with an incidence rate exceeding 50% in extensive-stage SCLC (ES-SCLC) (7). The blood-brain barrier/blood-tumor barrier (BBB/BTB) significantly impacts treatment options and clinical prognosis (6, 8).

Driver mutations are commonly found in NSCLC patients who are non-smokers or light smokers. Common mutation types include EGFR (exon 19 and exon 21 L858R mutation) mutation, ALK fusions, KRAS G12C mutation, BRAF V600E mutation, RET fusions, ROS1 fusions, HER2 mutation, NTRK fusions, and MET exon 14 skipping mutation (9, 10). Some NSCLC cases undergo histological transformation into SCLC, resulting in transformed SCLC (t-SCLC), which encompasses driver mutations commonly found in NSCLC (11). Targeted therapies for these mutations, such as tyrosine kinase inhibitors (TKIs), form the core treatment regimen. However, patients inevitably develop resistance, necessitating the exploration of novel therapeutic strategies.

The approval of immune checkpoint blockade (ICB) therapy has transformed the treatment landscape for lung cancer, delivering significant survival improvements, particularly for patients with advanced or refractory disease. ICB therapy restores anti-tumor immune responses by blocking inhibitory immune checkpoint molecules (such as PD-1/PD-L1 and CTLA-4), which are upregulated following T-cell activation. This promotes immune-mediated tumor clearance (1). In patients with BMs, ICIs primarily activate the peripheral immune system, enabling immune cells to cross the BBB and infiltrate metastatic brain lesions to exert cytotoxic effects. The discovery of the meningo-lymphatic system reveals a pathway through which immune cells can travel to and from the central nervous system, challenging the traditional concept of the brain’s “immune privilege” (12). ICIs, particularly PD-1/PD-L1 and CTLA-4 antibodies, have become the standard treatment for advanced NSCLC (13) and the standard first-line treatment for ES-SCLC (14) (**Table 1**). However, only a minority of patients achieve long-term benefits, and drug resistance remains a significant challenge in clinical practice.

**Table 1:** Clinical trials of ICIs for lung cancer.

Inhibitors	Test	Conclusion
PD-1 inhibitors		
Pembrolizumab	KEYNOTE-024	Pembrolizumab has been established as the standard first-line treatment for patients with advanced NSCLC who have a PD-L1 TPS of ≥50%
	KEYNOTE-042	Patients with advanced NSCLC confirmed to have low PD-L1 expression (TPS 1-49%) may derive an OS benefit from first-line treatment with pembrolizumab monotherapy
	KEYNOTE-189	Pembrolizumab combined with pemetrexed and platinum-based chemotherapy continues to deliver significant OS and PFS benefits in patients without EGFR/ALK mutations, treatment-naive metastatic non-squamous NSCLC, regardless of PD-L1 expression levels
	KEYNOTE-010	Pembrolizumab significantly prolonged overall survival in previously treated patients with PD-L1-positive (TPS ≥1%) advanced NSCLC, demonstrating superior safety compared to docetaxel, the benefit was particularly evident in the PD-L1 TPS ≥50% subgroup
	KEYNOTE-407	Pembrolizumab combined with carboplatin + paclitaxel/albumin-bound paclitaxel as first-line therapy for treatment-naive metastatic squamous NSCLC delivers durable OS, PFS, and PFS2 benefits, nearly doubling the 5-year OS rate, and demonstrates efficacy regardless of PD-L1 TPS expression levels
	KEYNOTE-091	Pembrolizumab significantly improves disease-free survival (DFS) in the overall population as adjuvant therapy for patients with completely resected stage IB-IIIA NSCLC, without requiring biomarker selection
	KEYNOTE-671	Combination of pembrolizumab with cisplatin-based chemotherapy during the perioperative period significantly improved OS and event-free survival compared with neoadjuvant chemotherapy alone in patients with resectable stage II/IIIA/IIIB NSCLC
	PePS2	Pembrolizumab can be safely administered to patients with PS2 status without increasing the risk of immune-related or other toxicities. Its efficacy outcomes are at least comparable to those in patients with PS0–1 status
	KEYNOTE-158/028	Pembrolizumab demonstrated durable antitumor activity in patients with recurrent/metastatic SCLC who had received two or more prior lines of therapy, with efficacy independent of PD-L1 expression status
	LIBRETTO-431	Selpercatinib delivers significant therapeutic benefits as first-line therapy for patients with advanced RET fusion-positive NSCLC, demonstrating superior efficacy compared to platinum-based chemotherapy with or without pembrolizumab. This confirms the critical importance of RET fusion testing in NSCLC diagnosis
Nivolumab	CheckMate 017/057	Nivolumab demonstrated a significant and durable 5-year survival benefit in patients with advanced NSCLC who progressed after first-line platinum-based chemotherapy, with a 5-year overall survival rate five times that of docetaxel, regardless of histological subtype or PD-L1 expression level
	CheckMate 227	Nivolumab plus ipilimumab as first-line therapy for metastatic NSCLC significantly improves 5-year survival rates regardless of tumor PD-L1 expression status, delivering durable clinical benefits that persist after treatment discontinuation, and this regimen demonstrates efficacy in both squamous and non-squamous subgroups
	CheckMate 9LA	Nivolumab plus Ipilimumab combined with two cycles of chemotherapy significantly improved OS, PFS, and objective response rate (ORR) in patients with advanced NSCLC compared to chemotherapy alone. This benefit was observed regardless of PD-L1 expression levels and tumor histology, establishing it as a new first-line treatment option for advanced NSCLC without EGFR/ALK mutations and has been approved in multiple countries
	CheckMate 816	Nivolumab combined with chemotherapy significantly improves perioperative event-free survival and pathological complete response in resectable NSCLC without compromising surgical feasibility
	CheckMate 817	Nivolumab combined with Ipilimumab demonstrated manageable safety and durable efficacy in ECOG PS 0-1 NSCLC patients, consistent with previous body weight-based dosing regimens. Treatment-related toxicity was manageable in special populations (including ECOG PS 2 and untreated BMs), yielding clinically meaningful long-term survival benefits
	CheckMate 331	In second-line treatment for recurrent SCLC, nivolumab monotherapy did not significantly improve overall survival but demonstrated longer duration of response and superior safety compared to chemotherapy.
	CheckMate 032	Nivolumab monotherapy for third-line or later treatment of SCLC delivers durable anti-tumor activity with favorable safety; combination with ipilimumab further improves long-term survival and demonstrates significantly superior efficacy compared to chemotherapy
	CheckMate-77T	Combination of Nivolumab with chemotherapy during the perioperative period significantly improves event-free survival and increases pathological response rates in patients with resectable NSCLC, without compromising surgical feasibility
Cemiplimab	EMPOWER-Lung 1	Cemiplimab monotherapy as first-line treatment for advanced NSCLC with PD-L1 expression ≥50% and no targetable mutations demonstrated sustained and significant survival benefits at 35 months follow-up (doubled overall survival, 50% improvement in PFS), with superior safety compared to chemotherapy
Serplulimab	ASTRUM-005	For patients with ES-SCLC who have not received prior systemic therapy, the combination of Sepelimumab with carboplatin plus etoposide as first-line treatment significantly improved OS, PFS, and tumor response rates compared with chemotherapy alone, while maintaining manageable safety
PD-L1 inhibitors		
Atezolizumab	IMpower010	Combination therapy with Atezolizumab following platinum-based adjuvant chemotherapy significantly improves DFS in patients with completely resected stage II-IIIA NSCLC, with particularly pronounced benefits in the PD-L1 TC≥1% subgroup and most notable gains in the PD-L1 TC≥50% subgroup
	IMpower110	Atezolizumab monotherapy has been shown to provide sustained benefits to OS and a superior safety profile in the first-line treatment of metastatic NSCLC patients who are treatment-naive and have high PD-L1 expression (TC ≥50% or IC ≥10%), as well as wild-type EGFR and ALK mutations
	IMpower130	Atezolizumab combined with carboplatin plus albumin-bound paclitaxel as first-line therapy for stage IV non-squamous NSCLC without EGFR/ALK mutations significantly improved OS and PFS compared to chemotherapy alone. The efficacy was not limited by PD-L1 expression levels
	IMpower150	The ABCP regimen achieved significant and sustained OS benefit in treatment-naive metastatic non-squamous NSCLC (regardless of EGFR/ALK mutation status); the ACP regimen conferred benefit in the PD-L1-positive subgroup
	POPLAR/OAK	LRP1B harmful mutations serve as a predictive biomarker for Atezolizumab efficacy: in non-squamous cancers, patients harboring this mutation are more likely to benefit from Atezolizumab monotherapy; in squamous cancers, patients without this mutation demonstrate relatively greater benefit from Atezolizumab monotherapy
	IMpower133	Atezolizumab combined with CP/ET significantly improved OS, PFS, and one-year survival rate in patients with ES-SCLC,demonstrating a favorable benefit-risk ratio without increasing the burden of treatment-related symptoms
	IFCT-1603	Atezolizumab monotherapy did not demonstrate significant efficacy as second-line therapy for recurrent SCLC
Durvalumab	PACIFIC	Durvalumab as consolidation therapy following concurrent chemoradiotherapy in patients with unresectable stage III NSCLC delivers significant and durable benefits in OS and PFS
	AEGEAN	Durvalumab full-cycle therapy (combined with platinum-based chemotherapy before surgery, followed by monotherapy after surgery) delivers significant survival benefits for patients with resectable stage II/III NSCLC. This regimen has become the new standard treatment for resectable stage II/III NSCLC without EGFR/ALK mutations
	CASPIAN	Durvalumab (with or without Tremelimumab) combined with platinum-based chemotherapy and etoposide as first-line treatment for ES-SCLC significantly improved OS. It also demonstrated clinical benefits in PFS, ORR, and response duration, with safety profiles comparable to conventional chemotherapy
	ADRIATIC	In patients with limited-stage small cell lung cancer (LS-SCLC) who remained disease-free after concurrent chemoradiotherapy, consolidation therapy with durvaluma, with or without Tremelimumab, effectively prolonged OS
Avelumab	JAVELIN Lung 200	In patients with advanced/relapsed NSCLC who progressed after platinum-based doublet therapy, avelumab demonstrated significant benefits in OS, PFS, and ORR across both PD-L1 high-expression subgroups (≥50% and ≥80%)
Adebrelimab	CAPSTONE-1	Adebrelimab in combination with carboplatin and etoposide significantly prolongs OS in Chinese patients with ES-SCLC
CTLA-4 inhibitors		
Ipilimumab	CheckMate 817	The conclusion is the same as stated earlier
	CheckMate 227/9LA	The conclusion is the same as stated earlier
	CheckMate 032	The conclusion is the same as stated earlier
Tremelimumab	CASPIAN	The conclusion is the same as stated earlier
	ADRIATIC	The conclusion is the same as stated earlier

## 2 The application of immune checkpoint inhibitors in lung cancer

### 2.1 Basic mechanism of immune checkpoint inhibitors

Immune checkpoint molecules usually act as negative regulators of immune cells immune function (15). The most common immunosuppressive target is the PD-1/PD-L1 pathway. PD-1 is widely expressed on immune cells, and is involved in both the adaptive immune system (T cells and B cells) and the innate immune system (natural killer (NK) cells and myeloid cells), especially activated T cells. Lung cancer cells express high levels of PD-L1, which binds to PD-1 on tumor-infiltrating lymphocytes (TILs) surfaces, induces the intracellular phosphorylation of the ITSM motif in PD-1 via T cell receptor (TCR) signaling, recruiting SHP-1/2 phosphatases to dephosphorylate key T cell activation molecules (CD3ζ, ZAP70), inhibiting T cell activation pathways such as PI3K/Akt. This impedes T cell proliferation and cytokine secretion, leading to a state of “exhaustion” where they lose their ability to kill tumor cells, ultimately promoting immune escape (16, 17). PD-1 is highly expressed in certain NK cell subsets, such as NK cells found in the peripheral blood of patients with cytomegalovirus and those found in patients with tumors. Binding to PD-L1 on tumor cells leads to NK cell exhaustion (18). PD-L1 is not only expressed on the surface of tumor cells but also exists in non-membrane forms within the cytoplasm and exosomes. It is more broadly expressed across various immune cells within the TME, including macrophages, dendritic cells, T cells, and B cells. The diverse forms and sources of PD-L1 are also key reasons for the suboptimal efficacy of immunotherapy. PD-L1 on T cells regulates immunity through bidirectional signaling (positively inhibiting effector T cells and inducing macrophage M2 polarization, while negatively regulating CD4^+^ T cell differentiation toward pro-tumor Th17 cells), and also maintains the survival of activated CD8^+^ T cells; PD-L1 on tumor-associated macrophages (TAMs), in addition to its traditional immunosuppressive role, can exhibits immunostimulatory effects in breast cancer, and its expression correlates with favorable prognosis in patients with multiple cancers; dendritic cells (DCs) suppress T cell activation via PD-L1, while simultaneously blocking both the PD-L1/PD-1 and CD80/CTLA-4 pathways through cis-binding to CD80, thereby further inhibiting T cell function; PD-L1 on regulatory B cells directly inhibits T cell activation and secretion of inhibitory cytokines (such as IL-10 and TGF-β), while also impeding the differentiation of CD4^+^ T cells into follicular helper T cells, thereby disrupting humoral immunity; the PD-L1 function of NK cells act as a self-protective mechanism to prevent excessive activation, its blockade significantly enhances their tumor-killing capacity (19).

Another clinically significant target is the CTLA-4 pathway, with 51%-87% of NSCLC tumors expressing CTLA-4. CTLA-4 is expressed during T cell activation and binds competitively to B7 molecules (a type of costimulatory molecule, that can provide the second signal necessary for T cell activation) on antigen-presenting cells (APCs) with CD28, blocking costimulatory signals and inhibiting T cell activation (1, 2, 15). Inhibiting these checkpoints enhances the immune system’s recognition and elimination of cancer cells.

TIGIT is a novel immune checkpoint that is co-expressed with PD-1 on TILs. TIGIT competes with CD226 for binding to the CD155/CD112 site, thereby counteracting activation signals, suppressing T cell function and inhibiting NK cell activation. *In vitro* studies have shown that blocking TIGIT increases the oncolytic potential of NK cells (18). Combined inhibition of PD-1 and TIGIT may enhance anti-tumor immune responses, particularly in NK cells (20).

**Figure 2:**
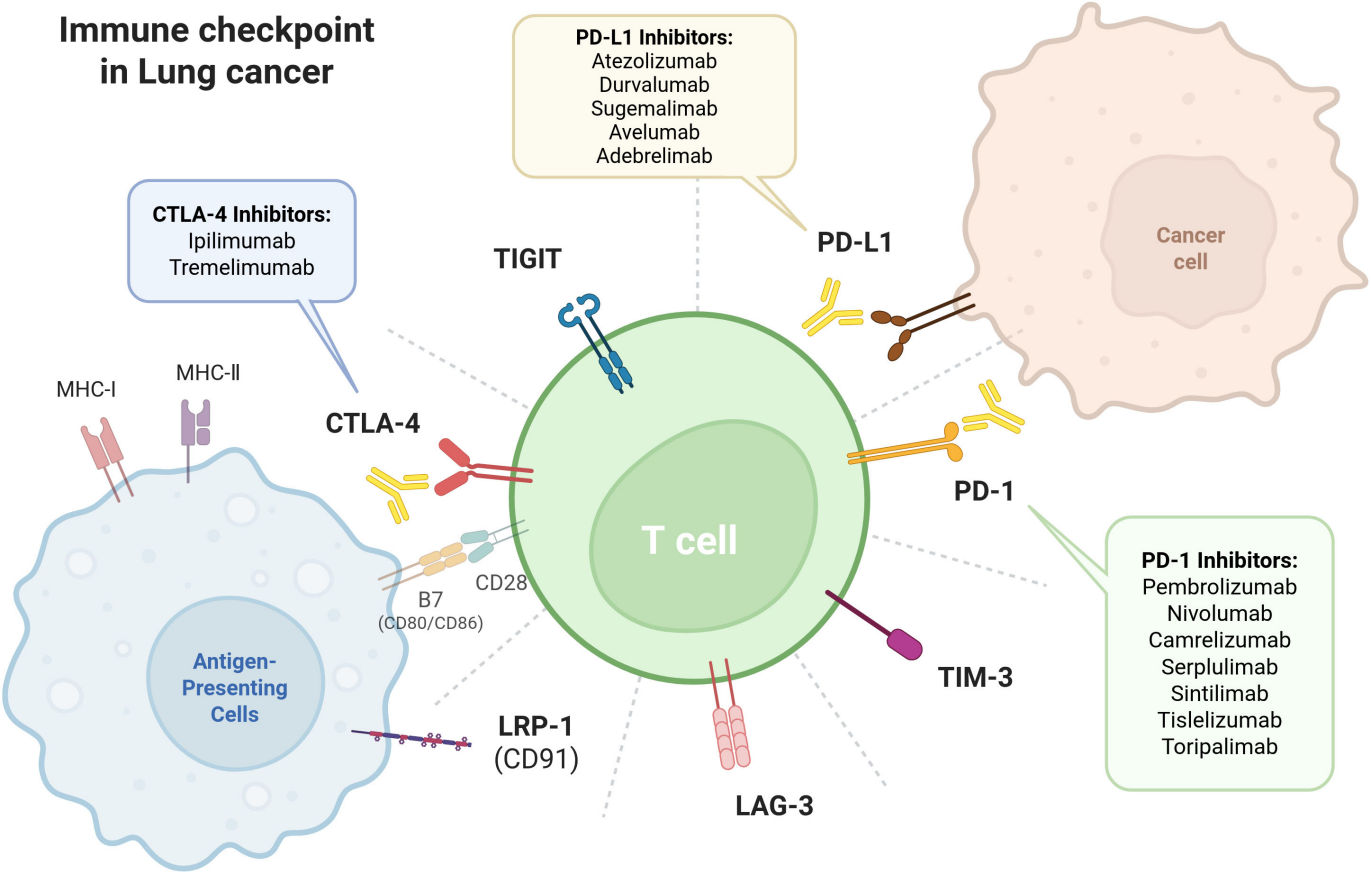
Immune checkpoints in lung cancer. Immune checkpoints commonly studied in lung cancer research are expressed on the surfaces of T cells, tumor cells, and APCs. Among these, the most extensively investigated PD-1/PD-L1 and CTLA-4 pathways have yielded multiple ICIs in clinical studies. Beyond the classic PD-1, PD-L1, and CTLA-4, numerous emerging immune checkpoints also hold promising application prospects.

ICB therapy restores anti-tumor immune responses by inhibiting immune checkpoints that are upregulated after T-cell activation (1, 21) (**Figure 2**). This reverses tumor immune escape and promotes immune-mediated tumor clearance. Certain combination regimens (e.g. PD-1 + TIGIT inhibitors) can activate both innate and adaptive immunity synergistically (20). The ICIs currently approved for clinical use primarily include anti-PD-1/PD-L1 and anti-CTLA-4 antibodies. Although they target the same immune checkpoint, different antibodies exhibit significant variations in molecular characteristic. Taking PD-1 antibodies as an example: Toripalimab binds to the FG loop of PD-1, pembrolizumab binds to the C’D loop, and nivolumab binds to the N-terminal loop, and Toripalimab has a binding affinity for human PD-1 that is 12-fold higher than that of pembrolizumab (22, 23). There are also differences in the clinical indications and irAEs between monoclonal antibodies of the same class but different species (22).

Treatment strategies vary according to the stage of disease progression. For limited-stage small cell lung cancer (LS-SCLC), radical chemoradiotherapy remains the core treatment, with the PD-L1 inhibitor Durvalumab approved by the FDA as consolidation therapy (**Table 1**. ADRIATIC trial), however, exploration of ICIs combined with chemoradiotherapy has yet to yield satisfactory results. For ES-SCLC, the standard first-line regimen is platinum-based chemotherapy with etoposide combined with PD-1/PD-L1 inhibitors. Serplulimab is the first approved PD-1 inhibitor (24) (**Table 1**. ASTRUM-005 trial). For operable, early-stage NSCLC (stages IB–IIIA), adjuvant immunotherapy with Atezolizumab or Pembrolizumab, administered after adjuvant chemotherapy, significantly prolongs disease-free survival. For unresectable locally advanced disease (stage III), Durvalumab (an anti-PD-L1 antibody) used as maintenance therapy following chemotherapy and radiotherapy markedly improves progression-free survival (PFS). For advanced NSCLC, treatment strategies vary based on PD-L1 expression. Patients with PD-L1 expression of at least 50% may consider monotherapy targeting PD-1/PD-L1. Those with PD-L1 expression of ≤50% should consider a combination of ICIs and platinum-based chemotherapy, as this yields superior outcomes. Dual ICIs combination therapy also outperforms chemotherapy alone (25). In asymptomatic patients with stable, small-volume BMs, ICIs demonstrate intracranial efficacy. Combining ICIs with radiotherapy results in a low incidence of radiation necrosis and may yield superior outcomes (26).

In NSCLC, eastern cooperative oncology group performance status (ECOG-PS) score is the most likely independent prognostic factor for evaluating ICI treatment, with significant differences in efficacy observed among patients with varying PS scores (13). ECOG-PS is the universal standard for assessing the physical functional status of patients in oncology. Physicians or nurses evaluate patients based on their physical activity capacity and their ability to perform daily activities independently: ECOG PS 0 patients are indistinguishable from healthy individuals; ECOG PS 1 patients experience mild tumor-related symptoms and slight functional limitations; ECOG PS 2 patients can still manage daily activities and walk independently, but are unable to work; ECOG PS 3 patients have limited self-care ability and require assistance with basic daily living activities; ECOG PS 4 patients experience profound physical decline and are unable to care for themselves or leave their bed; ECOG PS 5 indicates a terminal state (27). Patients with ECOG-PS score of 0–1 demonstrate the best response to ICI therapy, whereas those with ECOG-PS score of 3–4 show virtually no benefit from ICI treatment.

### 2.2 Monotherapy with immune checkpoint inhibitors

ICIs can enhance patients’ immune function, demonstrating superior efficacy and safety compared to traditional therapies (**Table 1**). For advanced NSCLC patients with PD-L1 expression of at least 50%, Pembrolizumab, Atezolizumab or Cemiplimab can be used as a first-line monotherapy (25). Furthermore, ICI monotherapy has been shown to be both effective and safe for patients with stable or asymptomatic BMs (12). Phase III trials such as CheckMate 227, CheckMate 017/057 and OAK have demonstrated that ICIs (e.g. Nivolumab and Atezolizumab) can significantly prolong overall survival (OS) and PFS in patients with NSCLC and BMs. This results in survival benefits that are comparable to, and sometimes even more pronounced than, those of patients without BMs (6). However, PD-L1 expression levels are not necessarily predictive of ICB efficacy, as patients with low or negative PD-L1 expression can also benefit.

For patients with EGFR/ALK mutations, monotherapy with ICIs, such as Nivolumab, Pembrolizumab or Atezolizumab, has not been shown to significantly improve survival rates compared to chemotherapy in second-line or later settings. Several clinical trials (e.g. OAK and CheckMate 057) have demonstrated limited efficacy. However, for KRAS-mutated patients, particularly those with the G12C subtype, ICI monotherapy may be somewhat effective. Responses to ICIs vary among other rare mutations, including BRAF, MET and ROS1, with overall response rates (ORR) remaining low (9).

### 2.3 Combination therapy with immune checkpoint inhibitors

Combination therapy involving dual ICIs (PD-1/PD-L1 + CTLA-4) produces synergistic effects by acting at different stages of T cell activation. CTLA-4 inhibitors function during the early stages of T cell proliferation to recruit T cell infiltration, while PD-1/PD-L1 inhibitors take effect during the late stages of T cell activation. Together, they improve the immunosuppressive microenvironment synergistically (2, 8). The CheckMate 227/9LA study demonstrated that, in advanced NSCLC, dual ICIs combination therapy ± short-term chemotherapy outperformed chemotherapy alone, independently of PD-L1 expression (2). This was the first regimen to confirm the efficacy of a dual ICIs combination therapy for treating NSCLC BMs. However, it has the drawback of increasing the risk of immune-related adverse events (12).

Chemotherapy can induce tumor cell death and release neoantigens, thereby enhancing the efficacy of immunotherapy. For patients with advanced NSCLC, a combination of Pembrolizumab and platinum-based chemotherapy can be used as a first-line treatment for those with PD-L1 expression of up to 50% (25). Studies such as KEYNOTE-189/407 and IMpower130 demonstrate that ICIs combined with chemotherapy significantly improve OS and PFS in both non-squamous and squamous NSCLC, establishing this combination as a standard first-line treatment regimen (2, 28). The synergistic effect of ICIs plus chemotherapy is most pronounced in patients with BMs, demonstrating greater OS and PFS improvements. This combination may enhance the anti-tumor efficacy of chemotherapy independently of PD-L1 expression (6). However, in patients with EGFR/ALK mutations, ICI plus chemotherapy did not significantly improve PFS or OS in second-line settings. For instance, the CheckMate 722 trial revealed no significant difference in PFS between Nivolumab plus chemotherapy and chemotherapy alone (9). The LIBRETTO-431 trial showed similar PFS results for chemotherapy with or without Pembrolizumab in patients with RET fusions (9).

Radiotherapy can damage tumor cell DNA, release tumor antigens, activate the cGAS-STING pathway and encourage the infiltration of immune cells. It can also induce PD-L1 expression, which synergizes with ICIs to enhance immune responses (8). Combining ICI with radiotherapy (especially stereotactic radiosurgery) improves local control rates, PFS and OS with favorable safety profiles. Concurrent or short-interval (<1 week) administration may yield superior response rates and survival benefits (6). The PACIFIC study established Durvalumab as a consolidation therapy following chemoradiotherapy for stage III NSCLC. Combining it with immunotherapy improves major response rates and survival, prolongs OS, and reduces new BMs. However, risks such as radiation pneumonitis, myocarditis and radiation necrosis require attention. Further prospective studies are needed to determine the optimal timing and dosage of combination therapy (2, 8).

Anti-angiogenic drugs combat ICIs resistance by normalizing blood vessels, thereby promoting the infiltration of immune cells and counteracting the TME’s immunosuppressive effects (29). The IMpower150 trial demonstrated that the combination of Atezolizumab + Bevacizumab + Carboplatin and Paclitaxel (ABCP) significantly prolonged OS and PFS in patients with EGFR/ALK mutations compared to the BCP regimen (9, 10). This regimen also reduced the incidence of new BMs (12). Bevacizumab may exert a corticosteroid-like effect by reducing peritumoral oedema without increasing the risk of intracranial hemorrhage (12). In the ATTLAS trial, ABCP significantly improved PFS and ORR in TKI-resistant patients with EGFR/ALK mutations, particularly in those with high PD-L1 expression (9).

The use of combination therapy involving ICIs and targeted drugs has also been studied. In patients with driver gene mutation-positive NSCLC, combination therapy with ICIs plus TKIs significantly improves ORR and disease control rate (DCR) compared to monotherapy with ICIs. However, the sequence of administration is associated with drug toxicity, necessitating further research on the optimal sequence and drug safety issues (10).

## 3 Drug resistance mechanisms affecting ICB therapy

**Figure 3:**
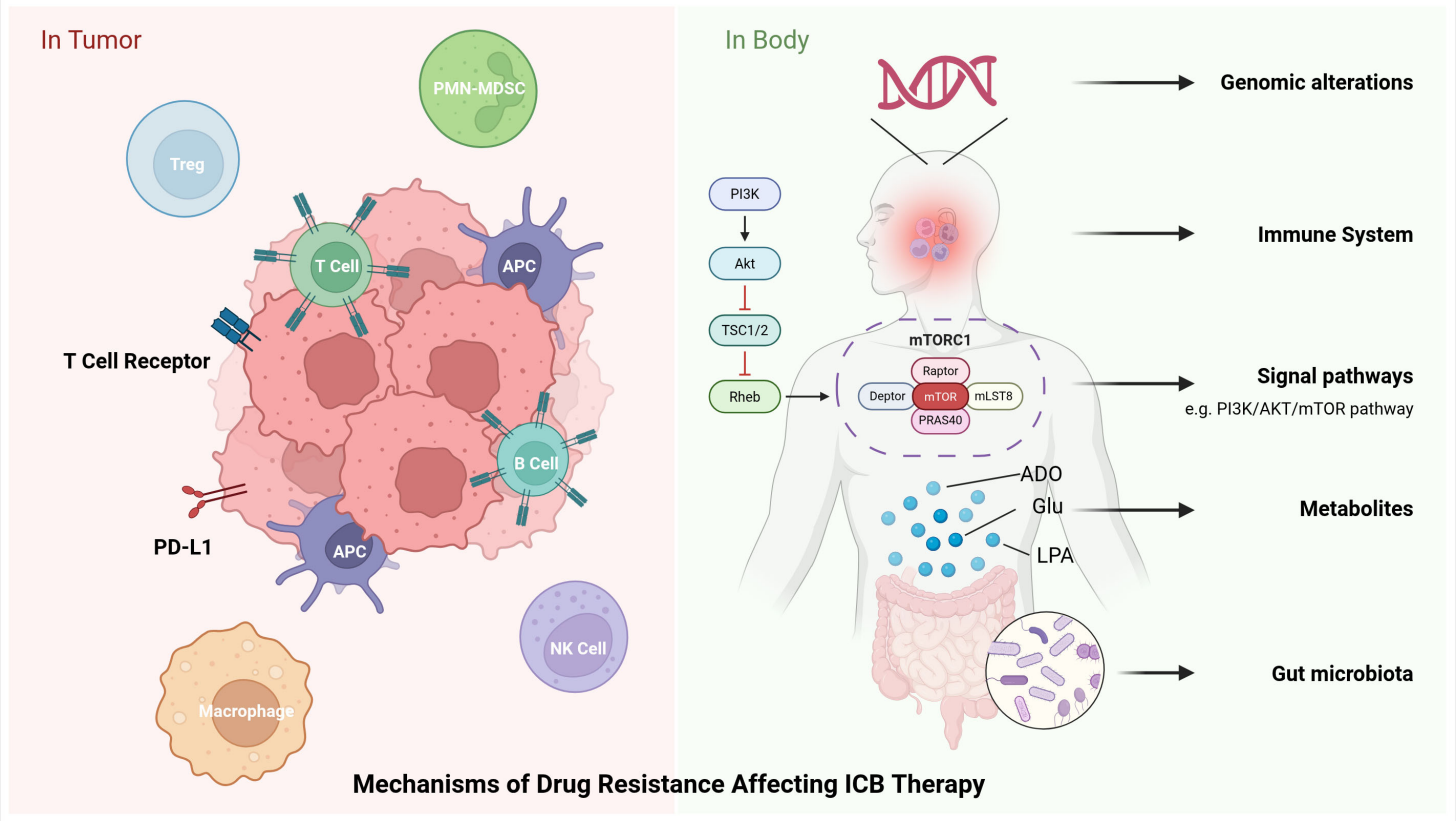
Mechanisms of resistance to ICB in lung cancer. Different tumor characteristics influence resistance to ICIs. Factors such as PD-L1 expression, TCR diversity, TILs in the immune microenvironment, immune-suppressive cells, and tumor heterogeneity all contribute to resistance. Additionally, genomic alterations, signaling pathways, impaired APMs, endogenous metabolites, and variations in gut microbiota within the human body also impact resistance to ICIs.

Although ICB therapy can significantly improve survival rates for patients with advanced lung cancer, only a subset of patients can achieve long-lasting responses, with most developing primary or acquired resistance. The mechanisms of ICB resistance are diverse and involve multiple levels, including intrinsic tumor cell mechanisms, the TME and the host immune system (**Figure 3**).

### 3.1 The impact of the tumor on immunotherapy

#### 3.1.1 Tumor microenvironment

ICB depends on immune cell infiltration within the TME. TILs are present in the TME, and a high density of these cells correlates with an improved response to ICIs. High T cell receptor diversity also predicts better efficacy, and mature tertiary lymphoid structures (TLS) show a positive correlation with ICIs response. B cells and plasma cells mature within TLS to enhance anti-tumor immunity (30). The TME also harbors diverse immunosuppressive cells. regulatory T cells (Tregs) suppress T cell function by expressing CTLA-4 and secreting TGF-β, while M2-type TAMs promote immunosuppression and are associated with PD-1 resistance. Myeloid-derived suppressor cells (MDSCs) accumulate in non-responders and inhibit T cell and NK cell function. MDSCs also negatively correlate with anti-PD-1 efficacy (31).

Tregs that are enriched in the NSCLC microenvironment are associated with a poor prognosis. Traditional ICBs are ineffective at eliminating these cells, so novel targeted strategies are required. MDSCs, particularly LOX1+ polymorphonuclear-MDSCs, are associated with ICIs resistance. Targeting CXCR2, IL-8 and FATP2 can reduce their immunosuppressive functions. M2 tumor-associated macrophages promote immunosuppression; CSF1R inhibitors can polarize them toward the M1 phenotype, thereby enhancing ICB efficacy (1).

SCLC exhibits a poor response to immunotherapy, primarily due to its TME, which is characterized by high expression of Tregs, low expression of MHC-I, high tumor heterogeneity and low immunogenicity (1, 20). SCLC cells secrete IL-15, which activates Tregs and suppresses CD4^+^ T cell proliferation. Tregs express CTLA-4 and PD-L1, which bind to APCs or T cells, activating immune checkpoint pathways and leading to tumor immune escape (20). The bispecific antibody Tarlatamab (targeting DLL3 and CD3) has shown promise in clinical trials. LSD1 inhibitors (e.g. Bomedemstat) can upregulate MHC-I expression, enhance T cell infiltration and improve ICB response (1).

NSCLC BMs exhibit an immunosuppressive state characterized by high expression of immunosuppressive molecules such as TGF-β, PD-L1 and IDO. There is lower T-cell infiltration than in primary tumors, with a near-complete absence of TILs and increased infiltration of tumor-associated macrophages, which further reinforces the immunosuppressive environment (8). TILs are crucial for the efficacy of ICIs, with their infiltration density and subtypes directly influencing the response to treatment. TIL density correlates with brain oedema severity, and low CD8^+^ TILs density is significantly associated with shorter OS. Innate immune cells, such as NK cells and tumor-associated macrophages, are active in BMs and may contribute to ICI response (6).

Patients with driver mutations, such as those in the EGFR and ALK genes, exhibit a “cold tumor” immune microenvironment characterized by low PD-L1 expression, minimal infiltration of CD8^+^ T cells, and a low TMB. This results in weak tumor immunogenicity and enhanced immune tolerance, ultimately leading to a poor response to ICIs, particularly when used as monotherapy (10).

#### 3.1.2 Tumor heterogeneity

The expression of PD-L1 and immune cell infiltration, such as TILs, as well as predictive biomarkers such as TMB, all exhibit spatial heterogeneity. There are significant variations in these factors across different tumor types and subtypes, as well as between primary and metastatic tumors in the same patient.

There is spatial and temporal heterogeneity in the expression of PD-L1, TILs and TMB between extracranial and intracranial lesions in NSCLC. Furthermore, PD-L1 expression in intracranial metastases can vary from case to case, potentially being higher or lower than in the primary tumor (6, 8).

SCLC exhibits significant immune heterogeneity, with treatment responses varying even between tumors of the same immune-inflammatory subtype. SCLC is classified into four subtypes based on the differential expression of transcription factors and inflammatory gene profiles: SCLC-A (ASCL1-high), SCLC-N (NEUROD1-high), SCLC-P (POU2F3-high) and SCLC-I (Inflamed) (32). Macrophage infiltration is a key factor influencing the efficacy of PD-L1 blockade, particularly in non-neuroendocrine (nonNE) phenotypes, where TAM enrichment induces immunosuppression. Of the four SCLC subtypes, SCLC-N and SCLC-A are “cold tumor” phenotypes, whereas SCLC-I-neuroendocrine (SCLC-I-NE) and SCLC-I-nonNE are both immune-inflammatory types characterized by high T-cell infiltration and elevated expression of antigen-presenting molecules. These two immune-inflammatory subtypes exhibit markedly different responses to PD-L1 blockade combined with chemotherapy. SCLC-I-NE demonstrates significant therapeutic benefit (median survival extended to 16.37 months), whereas SCLC-I-nonNE shows limited benefit (median survival of around 9.19 months) (33). Within the immunologically “hot tumor” category, tumors exhibiting high T-cell and low macrophage profiles (T-eff-high/TAM-low) demonstrated an optimal response to PD-L1 blockade, whereas those with high T-cell and high macrophage profiles (T-eff-high/TAM-high) showed a poor response. Consequently, the significant TAM enrichment in SCLC-I-nonNE may explain its suboptimal response to immunotherapy (33).

### 3.2 The impact of human variability on immunotherapy

The absence or low expression of PD-L1 is a common cause of drug resistance. The tumor immune microenvironment is classified into four types (T1-T4), with type T2 (PD-L1^+^, TIL^+^) being the most sensitive to ICIs. However, only around 17% of NSCLC patients fall into this category. The expression of PD-L1 on intratumoral immune cells (e.g. macrophages and DCs) may also influence treatment efficacy. In SCLC, the vast majority of tumor cells exhibit extremely low PD-L1 expression, and there is no clear correlation with ICI efficacy (14). Soluble PD-L1 serves as a prognostic marker, with high levels of expression correlating with poor outcomes (31).

Genomic alterations can lead to changes in the immune microenvironment. Tumor EGFR mutations result in low PD-L1 expression, low TMB and reduced T-cell infiltration, leading to a poor ICB response. Targeted therapy is therefore recommended as the primary treatment (1). STK11/LKB1 and KEAP1 mutations cause metabolic reprogramming and epigenetic silencing of the STING pathway. This reduces immune cell infiltration and results in a poor ICB response. Certain KRAS mutation subtypes (e.g. those with concurrent P53 or G12C mutations) exhibit a favorable ICI response, whereas G12D mutations may suppress immune responses and induce resistance (1, 31). Concurrent STK11/LKB1 or KEAP1 mutations confer a “cold tumor” phenotype with a poor ICB response. Deletion of the CDKN2A gene (chromosome 9p21.3) creates an immunosuppressive microenvironment associated with a “cold tumor” phenotype, low TIL infiltration and ICI resistance (1, 31). Deletion of Anti-Silencing Function 1A (ASF1A) enhances anti-PD-1 sensitivity (34).

Non-genomic signaling pathways also influence immune responses. Activation of CARM1 or ASCC3 can downregulate IFN signaling, leading to immune escape. Conversely, sustained IFN signaling can upregulate PD-L1, promoting acquired resistance (1). The Wnt/β-catenin pathway is associated with T cell exclusion. Upregulation of this pathway inhibits CCL4 secretion, reduces DC and CD8^+^ T cell infiltration, and results in a “cold tumor” phenotype. Targeting this pathway (e.g. using Porcupine inhibitors) can improve the response to ICB therapy (1, 31). The PI3K/AKT/mTOR pathway correlates with PD-L1 upregulation, and combining mTOR inhibitors may enhance ICI efficacy. The RAS/MAPK pathway drives PD-L1 expression and reduces TIL infiltration (31). Conversely, JAK1/2 inactivating mutations result in PD-L1 loss and diminished ICI response (35).

Impairment of the antigen presentation machinery (APM) can lead to resistance. β2M deficiency or mutation reduces MHC-I expression, resulting in ICB resistance. The downregulation or absence of HLA-I molecules affects immunopeptide diversity, thereby limiting T cell recognition (1, 31). In SCLC, resistance to ICIs induced by HLA-I deficiency can be overcome by activating IL-27/STAT3 to upregulate HLA-I expression, thereby enhancing ICI efficacy (36). HLA genotypes also influence ICI responses; high homozygosity at HLA-I loci reduces ICI sensitivity, whereas heterozygous HLA-I loci are associated with a better prognosis (31). Other non-classical HLA molecules (e.g. HLA-G and HLA-E) can suppress T cell function by binding to inhibitory receptors (31). Epigenetic modulators, such as HDAC/DNMT inhibitors, can upregulate APM and enhance ICB responses (1, 14).

In addition, metabolites within the human body can suppress the function of immune cells. Metabolites such as adenosine, glutamine and lysophosphatidic acid often affect immune cells (37). CD39/CD73 catalyses the conversion of ATP to adenosine, which suppresses T cell function via A2A/A2B receptors. Targeting this pathway (e.g. using CD73 inhibitors, A2A/A2B antagonists or PEG-ADA) can enhance ICB efficacy. The key metabolites of glutamine are vital for tumor cell proliferation, while its metabolic state directly influences immune cell function. Inhibiting glutaminase activity reduces tumor cells’ dependency on glutamine. Targeting glutaminase (e.g. with CB-839) or using glutamine antagonists (e.g. with DRP-104) can improve the response to ICB therapy. Lysophosphatidic acid (LPA) inhibits T cell migration and function via LPAR5/LPAR6. Targeting the autotaxin enzyme or LPAR5 enhances ICB efficacy. Furthermore, a secreted PD-L1 splice variant competitively binds to anti-PD-L1 monoclonal antibodies, thereby neutralizing their efficacy (37).

The composition of the gut microbiota can affect the efficacy of ICIs. For example, microbial species such as Akkermansia muciniphila and Bifidobacterium are associated with enhanced immune responses, whereas dysbiosis may diminish the efficacy of ICIs (31). Metabolites from microorganisms in the blood and respiratory tract may also serve as predictive biomarkers (30).

## 4 Individual challenges in immunotherapy

### 4.1 Immune checkpoint markers have limitations

The core biomarkers currently used in clinical screening to identify populations that will benefit from ICIs are PD-L1 expression levels and TMB. Specific genomic alterations can also predict the efficacy of ICIs.

PD-L1 expression occurs on tumor and immune cells, but its detection is influenced by factors such as the type of antibody used and the threshold criteria. Patients with NSCLC who have high PD-L1 expression demonstrate higher response rates to ICIs, yet the absence of a unified scoring standard, coupled with the fact that some PD-L1-negative patients still benefit from ICB, prevents PD-L1 expression from serving as a standalone basis for precision screening. In NSCLC patients with BMs, there is spatial heterogeneity between PD-L1 expression in BMs and primary tumors, with inconsistencies ranging from 14% to 26% (38). The predictive value of PD-L1 expression in BMs remains to be clarified. In contrast to NSCLC, PD-L1 expression in SCLC tumor cells ranges from 0% to 80%, primarily occurring in immune or stromal cells. Therefore, it cannot serve as a predictive biomarker for immunotherapy efficacy (5, 20).

TMB, which serves as an indirect indicator of tumor neoantigen abundance, remains difficult to standardize in detection methods (2). A high TMB (≥10 mut/Mb) positively correlates with an ICI response, particularly with dual ICIs combination therapy (2). This association is more pronounced in smoking-related lung cancer. BMs from NSCLC exhibit higher TMB than primary tumors, particularly lung adenocarcinoma BMs, which display the highest TMB levels of all cancer types (8). However, TMB fails to reflect dynamic changes in the immune microenvironment. Tumor heterogeneity leads to substantial variability in TMB measurements, and some patients respond even within low-TMB cohorts.

Novel immune checkpoints also have broad clinical application prospects. For example, inhibitory co-stimulatory molecules such as LAG-3, TIM-3 and TIGIT are highly expressed in drug-resistant tumors and are associated with PD-1 resistance (1, 31). LAG-3 can co-express with PD-1 to suppress T cell function, and it is currently approved for use in combination with PD-1 inhibitors to treat melanoma. TIM-3 and TIGIT are expressed in NSCLC and correlate with disease prognosis. TIM-3 antibodies have demonstrated favorable safety profiles in early trials, whereas TIGIT antibodies have failed to meet efficacy expectations in Phase III trials. VISTA (PD-1H, a PD-1 homologue) is primarily expressed on myeloid cells and, in NSCLC, on T cells. Its expression is pH-dependent and its function is complex, exhibiting both inhibitory and stimulatory effects that are potentially linked to the immunosuppressive microenvironment. CD91 (LRP-1) is expressed on APCs and participates in immune activation; its expression correlates with immunotherapy prognosis (31). Siglec-15 is primarily expressed on macrophages and exhibits mutual exclusion with PD-L1, making it a potential target for combination therapy (1).

Single biomarkers have limited predictive capability and may overlook intratumoral/intertumoral heterogeneity and dynamic evolution during treatment. Therefore, evaluation criteria should be established that incorporate multidimensional indicators, such as a combined assessment of immune cell infiltration, metabolic characteristics and epigenetic features.

### 4.2 Immune-related adverse events

**Figure 4:**
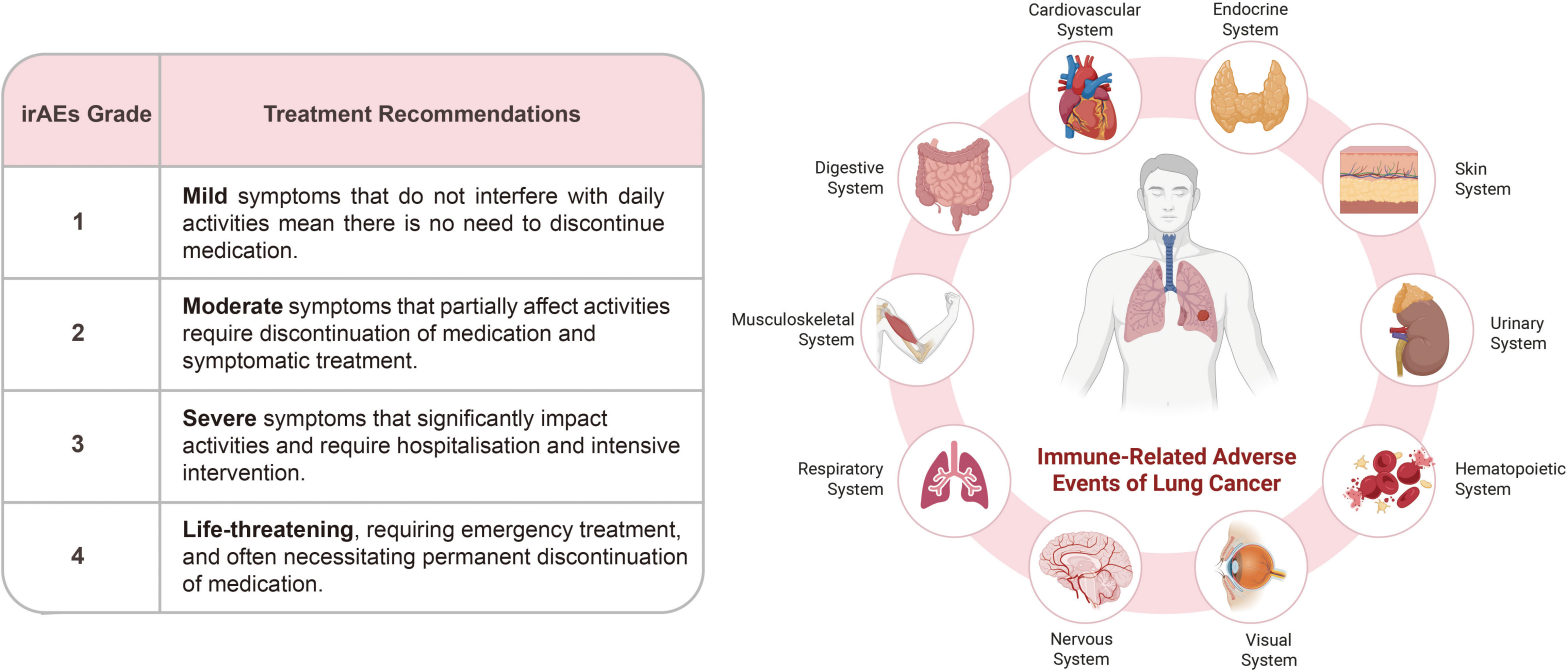
irAEs in lung cancer and their grading criteria. irAEs are unexpected occurrences in ICB therapy that affect multiple organs and systems in the body. Some irAEs have high incidence rates, enabling early intervention, while those with low incidence rates and a poor prognosis require particular attention during treatment monitoring. A graded, symptomatic management strategy categorizes irAEs into four levels to optimize drug usage.

ICIs may cause off-target inflammatory damage in lung cancer treatment by overactivating T cells or triggering immune responses against non-cancer antigens. Diseases caused by this type of damage are referred to as irAEs (**Figure 4**).

Respiratory diseases are the most common cause of treatment discontinuation due to irAEs in lung cancer patients. The most prevalent conditions are pneumonia and interstitial lung disease. Pneumonia, in particular, is the most dangerous respiratory irAE and can progress to acute respiratory distress syndrome and hypoxemia. Some patients also experience a low-grade fever. NSCLC patients are at a higher risk, which is closely linked to smoking history and underlying lung conditions (e.g. chronic obstructive pulmonary disease). Endocrine irAEs often cause irreversible damage that requires lifelong management, making them a key focus for long-term follow-up after ICI therapy. These primarily include thyroid dysfunction, hypophysitis, adrenal insufficiency and type 1 diabetes. Thyroid dysfunction is relatively common among these, with hypothyroidism occurring at a rate of 3.8%-13.2% and hyperthyroidism at 0.6%-8.0%. Skin irAEs are the most prevalent adverse events associated with ICI therapy, with rash and pruritus being the most frequent occurrences. They typically manifest as maculopapular eruptions or erythema, predominantly affecting the trunk and extremities. Severe irAEs, including Stevens-Johnson syndrome and toxic epidermal necrolysis, necessitate the permanent discontinuation of ICI treatment. Gastrointestinal irAEs have a relatively high incidence and primarily manifest as colitis, diarrhea and enteritis. This combination of symptoms is the most frequently observed irAE, with anti-CTLA-4 antibodies being associated with a significantly higher risk of more severe symptoms (29, 39).

Other irAEs are less common but have a worse prognosis. Cardiovascular irAEs have low incidence rates yet the highest fatality rates. Myocarditis is the core event, alongside pericarditis, arrhythmias and others. Symptoms are non-specific, which makes diagnosis challenging and prone to confusion with tumor-related cardiac injury. Early manifestations may be limited to palpitations, chest tightness and fatigue, all of which are easily overlooked. Progression may lead to chest pain, heart failure, malignant arrhythmias and sudden cardiac death. Urinary system irAEs primarily involve acute kidney injury, which is often accompanied by other systemic irAEs. The main manifestation is immune-mediated nephritis. Hematological irAEs are rare but severe, occurring in less than 1% of cases. These primarily include hemolytic anemia, immune thrombocytopenia, aplastic anemia, and neutropenia. Neurological irAEs occur at lower rates and include peripheral neuropathy, myasthenia gravis, encephalitis and aseptic meningitis, with an overall incidence of 3.8%–12%. The risk increases with combination ICI therapy. Musculoskeletal irAEs are relatively common and can be mistaken for tumor-related pain. These primarily include arthralgia, myalgia and inflammatory arthritis, with an incidence rate of up to 40% (29, 39).

Patients with a history of autoimmune diseases are more prone to irAEs. Although age and gender do not significantly influence irAEs incidence, elderly patients may be more vulnerable to ICI therapy due to immune senescence. The risk profile varies depending on the treatment regimen, whether it is monotherapy, dual-agent combinations or use in conjunction with chemotherapy. The type of ICIs and ethnicity can also influence the occurrence of irAEs. For instance, PD-L1 inhibitors are more likely to cause ocular surface disorders in ocular irAEs, while uveitis is more common with CTLA-4 inhibitors. Dual ICIs combination therapy (PD-1/PD-L1 + CTLA-4) are associated with a higher incidence of ophthalmoplegia. Ophthalmoplegia occurs significantly more frequently in Asian patients than in Caucasians, while uveitis occurs relatively more frequently in African Americans (40).

Additionally, blocking the PD-1/PD-L1 pathway may boost tuberculosis-specific T-cell responses, which could lead to the reactivation of latent tuberculosis (41). However, latent tuberculosis should not be considered an absolute contraindication to the use of ICIs. Screening is recommended for high-risk patients, with prophylactic anti-tuberculosis therapy administered when necessary.

Re-challenge with ICIs involves reintroducing the same class of ICI following discontinuation due to disease progression or irAEs. This is a viable strategy for some patients with advanced NSCLC, particularly if they have progressed on the previous agent or have manageable irAEs (39). irAEs are graded from 1 to 4 based on symptom severity, organ involvement, and impact on quality of life. Mainstream guidelines (CTCAE/ASCO/ESMO/NCCN/CSCO) recommend a “grade-specific symptomatic management” approach (29). Low grade (Grade 1-2): Most patients can continue or resume treatment after a temporary interruption. High grade (Grade 3-4): Most require permanent discontinuation. Some patients with endocrine adverse reactions (e.g. hypothyroidism or diabetes) may be able to continue ICI therapy with hormone replacement therapy or once the condition is under control (39, 42).

### 4.3 Variability in treatment outcomes across patient populations

The median age at lung cancer diagnosis exceeds 70 years (26). Elderly patients exhibit reduced immune function, diminished T-cell proliferation capacity and increased inhibitory signaling. During ICB therapy, factors such as organ damage and complications that are beyond control may arise, potentially compromising ICI efficacy. ICI monotherapy and combination chemotherapy have been shown to be effective in patients aged 65 years and over, particularly those with high PD-L1 expression. There is limited efficacy data for patients aged 75 years and over, especially regarding ICIs combination therapies (e.g. PD-1 + CTLA-4), which may be ineffective. Further clinical trials targeting elderly patients are recommended, incorporating CGA to inform personalized treatment strategies (26, 43).

Tumors in smokers exhibit high TMB and heightened immune activity within the TME. ICB therapy should be prioritized as the first-line treatment, as it has been shown to be particularly effective against smoking-related driver mutations (e.g. KRAS G12C), regardless of PD-L1 expression status. In contrast, tumors in non-smokers often exhibit low immunogenicity phenotypes (e.g. EGFR/ALK mutations) and demonstrate poor sensitivity to single-agent ICIs. Targeted therapy is therefore recommended as the first-line approach. Only patients with negative driver mutations and high PD-L1 expression/high TMB should consider single-agent ICB therapy. Otherwise, combination with other therapies (e.g. anti-angiogenic agents or chemotherapy) is necessary to enhance efficacy (1, 9).

Patients with a high BMI demonstrated superior outcomes to those with a low BMI during ICB therapy, with significantly longer PFS and OS (13).

ICIs are safe and effective for patients with well-controlled HIV infection, with no effect on viral load. In patients with well-controlled viral hepatitis (HBV/HCV), ICIs demonstrate favorable safety profiles and do not significantly increase hepatotoxicity. Prior to initiating ICB therapy, viral status should be screened, and liver function and viral load should be closely monitored during treatment (26).

Lung cancer patients with poor physical condition (ECOG-PS ≥2) often have a poor prognosis and face high toxicity risks with conventional chemotherapy. Due to their milder toxicity profile, ICB may serve as an alternative. However, most current ICI trials exclude these patients, so careful selection is necessary (15, 26).

In patients with NSCLC, sarcopenia is negatively correlated with the efficacy of ICIs and serves as a marker of poor prognosis (13). ICB therapy is not recommended for patients who have received a solid organ transplant, those with severe organ dysfunction, or those with severe autoimmune diseases.

## 5 Future directions and conclusion

ICB has emerged as a revolutionary immunotherapy that has transformed the treatment of lung cancer, particularly NSCLC, significantly. By blocking inhibitory signaling pathways such as PD-1/PD-L1 and CTLA-4, ICIs restore T-cell anti-tumor activity and reverse tumor immune escape. This achieves substantial prolongation of survival in patients with advanced, refractory lung cancer. ICIs are now integrated into every stage of lung cancer treatment, from early-stage adjuvant therapy and locally advanced consolidation therapy to first-line and subsequent-line treatments for advanced disease. They have become a vital part of precision medicine for lung cancer.

In clinical practice, ICB therapy necessitates a personalized approach that integrates multiple determinants, including tumor stage, PD-L1 expression, TMB, and the patient’s performance status. For resectable early-stage NSCLC, drugs such as Atezolizumab and Pembrolizumab, when used in adjuvant therapy, can significantly prolong disease-free survival. A neoadjuvant regimen combining Nivolumab with chemotherapy improves pathological complete response rates. For patients with unresectable, locally advanced NSCLC, consolidation therapy with Durvalumab following chemoradiation markedly extends both overall and PFS. Treatment strategies for advanced NSCLC focus on PD-L1 expression levels. For patients with advanced NSCLC and high PD-L1 expression (≥50%), PD-1/PD-L1 monotherapy has become the standard first-line treatment. For patients with low or negative PD-L1 expression, ICIs combined with chemotherapy or dual ICIs combination therapy can significantly enhance efficacy. In patients with BMs, ICIs demonstrate intracranial activity, particularly when combined with radiotherapy or anti-angiogenic agents, thereby improving local control and survival outcomes further. However, ICIs demonstrate limited efficacy in patients with driver gene mutations, such as EGFR/ALK positivity, for whom targeted therapy remains the primary treatment option. However, some studies suggest that combining ICIs with anti-angiogenic therapy or chemotherapy could be beneficial in these cases.

Since 2019, the combination therapy of etoposide plus platinum-based chemotherapy (EP) and ICIs has shown promising applications in improving survival outcomes for patients with SCLC (44). Building on this evidence, PD-L1 inhibitors (Atezolizumab and Durvalumab) or PD-1 inhibitors (Serplulimab), combined with chemotherapy, have now become the standard first-line treatment for extensive-stage SCLC (ES-SCLC), significantly improving OS. In limited-stage SCLC (LS-SCLC), Durvalumab can be used as consolidation therapy after chemoradiotherapy. Currently, the humanized monoclonal antibodies targeting PD-L1, Atezolizumab and Durvalumab, have been approved in multiple countries for the first-line treatment of small cell lung cancer (45, 46). However, immunotherapy for SCLC also faces numerous challenges. For instance, PD-L1 upregulation is observed in only a small subset of patients, indicating that immune evasion mechanisms play a more dominant role in the majority of cases. This phenomenon is partly attributed to the overexpression of B7-H3, a B7 family immune checkpoint ligand. B7-H3 is overexpressed in approximately 65% of SCLC cases, thereby significantly inhibiting T-cell activation and proliferation, and enhancing the potential for tumor evasion from immune surveillance (47, 48). Based on this, to overcome the challenge of immune evasion in tumor treatment, emerging novel immunotherapeutic strategies are currently in the stage of rapid clinical translation. For example, antibody-drug conjugates (ADCs) targeting the highly expressed B7-H3, such as YL201, have achieved an objective response rate of up to 63.9% in previously treated patients with extensive-stage SCLC, providing robust evidence for overcoming immune resistance (49). Tarlatamab, which has already been approved for market, is a bispecific T-cell engager (BiTE) designed to target DLL3. It has achieved the effect of effectively killing tumor cells through the forced activation of T cells (50). Even more advanced is the CAR-T cell therapy targeting B7-H3, which holds promise for bringing durable remission effects to patients with SCLC (51). Furthermore, the strategy of combining oncolytic viruses with ICIs is also expected to convert cold tumors into hot tumors, thereby further enhancing the efficacy of immunotherapy (52). Additionally, the continuously discovered cancer-associated molecules closely related to SCLC are providing new targets and decision-making bases for biomarker-guided precision therapy, offering potential new directions for overcoming immunotherapy resistance in SCLC.

However, the widespread application of ICIs still faces multiple challenges. Resistance mechanisms are complex and diverse, involving the TME (e.g. immune-suppressive cell infiltration and the absence of TLS), intrinsic tumor characteristics (e.g. defects in antigen presentation mechanisms and abnormal signaling pathways) and host factors (e.g. metabolic reprogramming and gut microbiota dysbiosis). In terms of biomarkers, although PD-L1 expression and TMB are used to predict ICI efficacy, they are not standardized, have limited predictive value, and exhibit spatiotemporal heterogeneity, particularly with regard to inconsistent expression within BMs. Consequently, future efforts should focus on developing integrated predictive models that combine genomic, immune microenvironmental, and metabolic characteristics.

irAEs are a significant concern in ICI therapy as they can affect multiple organ systems, including the skin, gastrointestinal tract, endocrine system, lungs and heart. While most irAEs can be managed with corticosteroids and symptomatic treatment, severe cases may require the drug to be discontinued permanently. There are significant variations in response to ICIs across different populations (e.g. elderly patients, smokers and those with differing performance statuses), which highlights the importance of making treatment decisions on a case-by-case basis, considering the patient’s underlying conditions, immune status and tumor biology.

In the future, lung cancer immunotherapy must evolve toward personalized precision medicine. This will involve developing novel ICIs (such as TIGIT, LAG-3 and TIM-3) and combination strategies, utilizing multi-omics technologies (e.g. single-cell sequencing, spatial transcriptomics and metabolomics) to analyze tumor immune microenvironment heterogeneity in depth, leveraging artificial intelligence and radiomics to predict treatment response early on and monitor it dynamically, overcoming resistance through personalized combination therapies (e.g. ICIs combined with targeted therapy, radiotherapy and metabolic modulators) and optimizing biomarker detection systems to advance the clinical practice of precision immunotherapy.

In summary, ICIs have made significant progress in the treatment of lung cancer. However, drug resistance, adverse reactions and therapeutic heterogeneity remain significant scientific and clinical challenges. By conducting ongoing basic and clinical research, fostering multidisciplinary collaboration, and developing personalized treatment strategies, we can improve long-term survival rates and enhance the quality of life for lung cancer patients. This will usher in a new era of greater precision and safety in tumor immunotherapy.

## References

[1] Konen JM Wu H Gibbons DL . Immune checkpoint blockade resistance in lung cancer: emerging mechanisms and therapeutic opportunities. Trends Pharmacol Sci. (2024) 45:520–36. doi: 10.1016/j.tips.2024.04.006. PMID: 38744552 PMC11189143

[2] Tang S Qin C Hu H Liu T He Y Guo H . Immune checkpoint inhibitors in non-small cell lung cancer: progress, challenges, and prospects. Cells. (2022) 11(3):320. doi: 10.3390/cells11030320. PMID: 35159131 PMC8834198

[3] Peifer M Fernandez-Cuesta L Sos ML George J Seidel D Kasper LH . Integrative genome analyses identify key somatic driver mutations of small-cell lung cancer. Nat Genet. (2012) 44:1104–10. doi: 10.1038/ng.2396. PMID: 22941188 PMC4915822

[4] Henriques GT Barbieri de Souza C Aguiar PN . Predictive genetic biomarkers in immune checkpoint inhibitors for non-small-cell lung cancer. Immunotherapy. (2022) 14:249–57. doi: 10.2217/imt-2021-0175. PMID: 35076285

[5] Kim SY Park HS Chiang AC . Small cell lung cancer: a review. JAMA. (2025) 333:1906–17. doi: 10.1001/jama.2025.0560, PMID: 40163214

[6] Wei Y Xu Y Wang M . Immune checkpoint inhibitors for the treatment of non-small cell lung cancer brain metastases. Chin Med J (Engl). (2023) 136:1523–31. doi: 10.1097/cm9.0000000000002163. PMID: 37106555 PMC10325750

[7] Wang C Mu S Yang X Li L Tao H Zhang F . Outcome of immune checkpoint inhibitors in patients with extensive-stage small-cell lung cancer and brain metastases. Front Oncol. (2023) 13:1110949. doi: 10.3389/fonc.2023.1110949. PMID: 37213269 PMC10196483

[8] Xiao G Liu Z Gao X Wang H Peng H Li J . Immune checkpoint inhibitors for brain metastases in non-small-cell lung cancer: from rationale to clinical application. Immunotherapy. (2021) 13:1031–51. doi: 10.2217/imt-2020-0262. PMID: 34231370

[9] Huber RM Kauffmann-Guerrero D . Immune checkpoint inhibitors in driver mutation-positive nonsmall cell lung cancer. Curr Opin Oncol. (2025) 37:35–9. doi: 10.1097/cco.0000000000001110. PMID: 39526694

[10] Lee J Ahn MJ . Immune checkpoint inhibitors in driver mutation-positive nonsmall cell lung cancer: is there a role? Curr Opin Oncol. (2021) 33:64–72. doi: 10.1097/cco.0000000000000698. PMID: 33186183

[11] Sivakumar S Moore JA Montesion M Sharaf R Lin DI Colon CI . Integrative analysis of a large real-world cohort of small cell lung cancer identifies distinct genetic subtypes and insights into histologic transformation. Cancer Discov. (2023) 13:1572–91. doi: 10.1158/2159-8290.cd-22-0620. PMID: 37062002 PMC10326603

[12] Yan X Qu F Zhou Y . Progress of immune checkpoint inhibitors therapy for non-small cell lung cancer with brain metastases. Lung Cancer. (2023) 184:107322. doi: 10.1016/j.lungcan.2023.107322. PMID: 37611495

[13] Kaira K Imai H Mouri A Yamaguchi O Kagamu H . Clinical effectiveness of immune checkpoint inhibitors in non-small-cell lung cancer with a poor performance status. Med (Kaunas). (2021) 57(11):1273. doi: 10.3390/medicina57111273. PMID: 34833490 PMC8618581

[14] Papavassiliou KA Stravopodis DJ Gogou VA Papavassiliou AG . Cracking the code of immunotherapy resistance in SCLC: molecular insights and emerging solutions. Am J Respir Crit Care Med. (2026) 23:aamaf124. doi: 10.1093/ajrccm/aamaf124. PMID: 41738120 PMC13253033

[15] Glode AE May MB . Immune checkpoint inhibitors: significant advancements in non-small cell lung cancer treatment. Am J Health Syst Pharm. (2021) 78:769–80. doi: 10.1093/ajhp/zxab041. PMID: 33580648

[16] Ortega MA Boaru DL De Leon-Oliva D Fraile-Martinez O Garcia-Montero C Rios L . PD-1/PD-L1 axis: implications in immune regulation, cancer progression, and translational applications. J Mol Med (Berl). (2024) 102:987–1000. doi: 10.1007/s00109-024-02463-3. PMID: 38935130

[17] Li Z Wang T Liu J Qi W Lv Q Xu Y . The mechanism and research progress of PD-1/PD-L1 on immune escape of lung cancer. Transl Cancer Res. (2025) 14:6041–51. doi: 10.21037/tcr-2025-230. PMID: 41158269 PMC12554497

[18] Fang F Xiao W Tian Z . NK cell-based immunotherapy for cancer. Semin Immunol. (2017) 31:37–54. doi: 10.1016/j.smim.2017.07.009. PMID: 28838796

[19] Hu L Sun C Yuan K Yang P . Expression, regulation, and function of PD-L1 on non-tumor cells in the tumor microenvironment. Drug Discov Today. (2024) 29:104181. doi: 10.1016/j.drudis.2024.104181. PMID: 39278561

[20] Li L Liang Y Yu M Zhao L Mei Q Yu Y . Advances in immune checkpoint inhibitors therapy for small cell lung cancer. Cancer Med. (2023) 12:11097–106. doi: 10.1002/cam4.5659. PMID: 36880420 PMC10242320

[21] Khan M Arooj S Wang H . NK cell-based immune checkpoint inhibition. Front Immunol. (2020) 11:167. doi: 10.3389/fimmu.2020.00167. PMID: 32117298 PMC7031489

[22] Parvez A Choudhary F Mudgal P Khan R Qureshi KA Farooqi H . PD-1 and PD-L1: architects of immune symphony and immunotherapy breakthroughs in cancer treatment. Front Immunol. (2023) 14:1296341. doi: 10.3389/fimmu.2023.1296341. PMID: 38106415 PMC10722272

[23] Lorch JH Stein S Edelman MJ . Are all programmed cell death protein 1 inhibitors the same? Front Oncol. (2025) 15:1535030. doi: 10.3389/fonc.2025.1535030. PMID: 40027132 PMC11867946

[24] Behrouzi R Blackhall F . State of the art in treatment of small cell lung cancer. Ther Adv Med Oncol. (2025) 17:17588359251363518. doi: 10.1177/17588359251363518. PMID: 40964483 PMC12437200

[25] Gargalionis AN Papavassiliou KA Papavassiliou AG . Immune checkpoint inhibitors in non-small cell lung cancer (NSCLC) treatment: quo vadis? Int J Mol Sci. (2024) 25(12):6309. doi: 10.3390/ijms25126309. PMID: 38928013 PMC11203558

[26] Escoin-Perez C Blasco S Juan-Vidal O . Immune checkpoint inhibitors in special populations. A focus on advanced lung cancer patients. Lung Cancer. (2020) 144:1–9. doi: 10.1016/j.lungcan.2020.03.026. PMID: 32278215

[27] Neeman E Gresham G Ovasapians N Hendifar A Tuli R Figlin R . Comparing physician and nurse Eastern Cooperative Oncology Group Performance Status (ECOG-PS) ratings as predictors of clinical outcomes in patients with cancer. Oncologist. (2019) 24:e1460-e1466. doi: 10.1634/theoncologist.2018-0882. PMID: 31227648 PMC6975959

[28] Jeon H Wang S Song J Gill H Cheng H . Update 2025: management of non-small-cell lung cancer. Lung. (2025) 203:53. doi: 10.1007/s00408-025-00801-x. PMID: 40133478 PMC11937135

[29] Gang X Yan J Li X Shi S Xu L Liu R . Immune checkpoint inhibitors rechallenge in non-small cell lung cancer: current evidence and future directions. Cancer Lett. (2024) 604:217241. doi: 10.1016/j.canlet.2024.217241. PMID: 39260670

[30] Yao J Lin X Zhang X Xie M Ma X Bao X . Predictive biomarkers for immune checkpoint inhibitors therapy in lung cancer. Hum Vaccin Immunother. (2024) 20:2406063. doi: 10.1080/21645515.2024.2406063. PMID: 39415535 PMC11487980

[31] Zhou K Li S Zhao Y Cheng K . Mechanisms of drug resistance to immune checkpoint inhibitors in non-small cell lung cancer. Front Immunol. (2023) 14:1127071. doi: 10.3389/fimmu.2023.1127071. PMID: 36845142 PMC9944349

[32] Gay CM Stewart CA Park EM Diao L Groves SM Heeke S . Patterns of transcription factor programs and immune pathway activation define four major subtypes of SCLC with distinct therapeutic vulnerabilities. Cancer Cell. (2021) 39:346–60:e7. doi: 10.1016/j.ccell.2020.12.014. PMID: 33482121 PMC8143037

[33] Nabet BY Hamidi H Lee MC Banchereau R Morris S Adler L . Immune heterogeneity in small-cell lung cancer and vulnerability to immune checkpoint blockade. Cancer Cell. (2024) 42:429–43:e4. doi: 10.1016/j.ccell.2024.01.010. PMID: 38366589

[34] Li F Huang Q Luster TA Hu H Zhang H Ng WL . *In vivo* epigenetic CRISPR screen identifies Asf1a as an immunotherapeutic target in Kras-mutant lung adenocarcinoma. Cancer Discov. (2020) 10:270–87. doi: 10.1158/2159-8290.cd-19-0780. PMID: 31744829 PMC7007372

[35] Shin DS Zaretsky JM Escuin-Ordinas H Garcia-Diaz A Hu-Lieskovan S Kalbasi A . Primary resistance to PD-1 blockade mediated by JAK1/2 mutations. Cancer Discov. (2017) 7:188–201. doi: 10.1158/2159-8290.cd-16-1223. PMID: 27903500 PMC5296316

[36] Carbotti G Nikpoor AR Vacca P Gangemi R Giordano C Campelli F . IL-27 mediates HLA class I up-regulation, which can be inhibited by the IL-6 pathway, in HLA-deficient small cell lung cancer cells. J Exp Clin Cancer Res. (2017) 36:140. doi: 10.1186/s13046-017-0608-z. PMID: 29020964 PMC5637329

[37] Gong B Kiyotani K Sakata S Nagano S Kumehara S Baba S . Secreted PD-L1 variants mediate resistance to PD-L1 blockade therapy in non-small cell lung cancer. J Exp Med. (2019) 216:982–1000. doi: 10.1084/jem.20180870. PMID: 30872362 PMC6446862

[38] Mansfield AS Aubry MC Moser JC Harrington SM Dronca RS Park SS . Temporal and spatial discordance of programmed cell death-ligand 1 expression and lymphocyte tumor infiltration between paired primary lesions and brain metastases in lung cancer. Ann Oncol. (2016) 27:1953–8. doi: 10.1093/annonc/mdw289. PMID: 27502709 PMC5035793

[39] Tang LB Peng YL Chen J Li JT Zheng MM Wu L . Rechallenge with immune-checkpoint inhibitors in patients with advanced-stage lung cancer. Nat Rev Clin Oncol. (2025) 22:546–65. doi: 10.1038/s41571-025-01029-7. PMID: 40490476

[40] Zhou L Wei X . Ocular immune-related adverse events associated with immune checkpoint inhibitors in lung cancer. Front Immunol. (2021) 12:701951. doi: 10.3389/fimmu.2021.701951. PMID: 34504488 PMC8421677

[41] Haddad FG Kattan C Kattan J . Should immune checkpoint inhibitors be contraindicated in lung cancer patients with latent tuberculosis? Immunotherapy. (2020) 12:759–62. doi: 10.2217/imt-2020-0069. PMID: 32517560

[42] Lee E Jang JY Yang J . Uncommon adverse events of immune checkpoint inhibitors in small cell lung cancer: a systematic review of case reports. Cancers (Basel). (2024) 16(10):1896. doi: 10.3390/cancers16101896. PMID: 38791974 PMC11119772

[43] Wang L Zhou J Yu X Su C . Immune checkpoint inhibitors in elderly patients with lung cancer: evidence from phase 3 trials. Curr Opin Oncol. (2024) 36:35–43. doi: 10.1097/cco.0000000000001006. PMID: 37975311

[44] Megyesfalvi Z Gay CM Popper H Pirker R Ostoros G Heeke S . Clinical insights into small cell lung cancer: tumor heterogeneity, diagnosis, therapy, and future directions. CA: A Cancer J For Clin. (2023) 73:620–52. doi: 10.3322/caac.21785. PMID: 37329269

[45] Frampton JE . Atezolizumab: a review in extensive-stage SCLC. Drugs. (2020) 80:1587–94. doi: 10.1007/s40265-020-01398-6. PMID: 32990939

[46] Al-Salama ZT . Durvalumab: a review in extensive-stage SCLC. Targeted Oncol. (2021) 16:857–64. doi: 10.1007/s11523-021-00843-0. PMID: 34731446 PMC8648650

[47] Zhong J Jie G Qin H Li H Chen N Aerxiding P . Immunotherapy for small cell lung cancer: current challenges and prospects. Exp Hematol Oncol. (2025) 14:130. doi: 10.1186/s40164-025-00720-w. PMID: 41194225 PMC12590821

[48] Zugazagoitia J Osma H Baena J Ucero AC Paz-Ares L . Facts and hopes on cancer immunotherapy for small cell lung cancer. Clin Cancer Research: Off J Am Assoc For Cancer Res. (2024) 30:2872–83. doi: 10.1158/1078-0432.ccr-23-1159. PMID: 38630789

[49] Ma Y Yang Y Huang Y Fang W Xue J Meng X . A B7H3-targeting antibody-drug conjugate in advanced solid tumors: a phase 1/1b trial. Nat Med. (2025) 31:1949–57. doi: 10.1038/s41591-025-03600-2. PMID: 40082695 PMC12176648

[50] Ji K Guo L Zuo D Feng M Chen X Zhao Z . Harnessing delta-like ligand 3: bridging biomarker discovery to next-generation immunotherapies in refractory small cell lung cancer. Front Immunol. (2025) 16:1592291. doi: 10.3389/fimmu.2025.1592291. PMID: 40496850 PMC12149116

[51] Kristmann B Werchau N Suresh L Pezzuto EL Scheuermann S Krost S . Targeting CD276 with Adapter-CAR T-cells provides a novel therapeutic strategy in small cell lung cancer and prevents CD276-dependent fratricide. J Hematol Oncol. (2025) 18:76. doi: 10.1186/s13045-025-01729-8. PMID: 40722088 PMC12305915

[52] Sun L Zhao Q Miao L . Combination therapy with oncolytic viruses for lung cancer treatment. Front Oncol. (2025) 15:1524079. doi: 10.3389/fonc.2025.1524079. PMID: 40248194 PMC12003109

